# Chronic Infection of Domestic Cats with Feline Morbillivirus, United States

**DOI:** 10.3201/eid2204.151921

**Published:** 2016-04

**Authors:** Claire R. Sharp, Sham Nambulli, Andrew S. Acciardo, Linda J. Rennick, J. Felix Drexler, Bertus K. Rima, Tracey Williams, W. Paul Duprex

**Affiliations:** Tufts University Cummings School of Veterinary Medicine, North Grafton, Massachusetts, USA (C.R. Sharp);; Boston University School of Medicine, Boston, Massachusetts, USA (S. Nambulli, A.S. Acciardo, L.J. Rennick, W.P. Duprex);; University of Bonn Medical Centre, Bonn, Germany (J.F. Drexler);; German Center for Infection Research, Bonn-Cologne Germany (J.F. Drexler);; The Queen’s University of Belfast School of Medicine, Dentistry, and Biomedical Sciences, Belfast, Northern Ireland (B.K. Rima);; Zoetis LLC, Kalamazoo, Michigan, USA (T. Williams)

**Keywords:** feline morbillivirus, FeMV, tubulointerstitial nephritis, chronic kidney disease, CKD, paramyxoviruses, viruses, United States, cats

**To the Editor:** Feline morbillivirus (FeMV) was first reported in Hong Kong and mainland China in 2012 (*1*) and has been associated with tubulointerstitial nephritis, the histopathologic correlate of idiopathic chronic kidney disease (CKD); however, this association has not been proven by studies in FeMV-naive animals. In 2013, phylogenetically related strains were found in Japan, indicating broader geographic distribution in Asia (*2*). The lack of complete genome sequences for strains from other regions prevents assessment of the clinical relevance and genetic diversity of FeMV. Classical morbilliviruses, such as measles and canine distemper viruses, have a global distribution, suggesting that FeMV might be present elsewhere in the world (*3*). To confirm the presence of FeMV and assess its genetic diversity and infection patterns in the United States, we collected and analyzed urine samples from domestic cats.

We generated amplicons from 10 (3%) of 327 samples; 3 samples were from cats with CKD and 7 from cats without CKD. Sequencing results confirmed that these 493 bp amplicons correspond to unique strains of FeMV (*1*). FeMV^US1^ is 97% similar in the L gene amplicon sequence to FeMV^776U ^(*1*), whereas FeMV^US5^ is only 85% identical, making it very different to all previously identified FeMVs. We used these sequences to develop a pan-US primer set, priFeMV^US^panL+ and priFeMV^US^panL−, to amplify a highly conserved region (460 bp) of the L gene of the US strains (Technical Appendix Table 1). The results of these analyses demonstrated that FeMV is present outside of Asia.

In October 2013, we obtained the initial FeMV^US1^-positive sample from a healthy 4-year-old male domestic shorthair cat (animal 0213). Approximately 15 months later, we obtained a follow-up urine sample from the still healthy cat, performed reverse transcription PCR (RT-PCR), and generated amplicons (Technical Appendix Figure 1, panel A). Amplification and sequencing of the hemagglutinin (H) gene from the 2015 sample indicated that it was identical to that from the 2013 sample, suggesting that the cat was chronically infected. We developed a quantitative RT-PCR test by using L gene primers and a real-time probe (Technical Appendix Table 2). Results indicated stable and comparable virus loads: 9.8 × 10^4^ copies/mL in 2013 versus 7.8 × 10^4^ copies/mL in 2015. This finding corroborates the view that cats can be chronically infected with FeMV and that the virus is persistently shed in urine.

We used primers to generate cDNA from clinical material and then determined the complete genome sequence of FeMV^US1^ (GenBank accession no. KR014147) by using RT-PCR and rapid amplification of cDNA ends. The major morbillivirus surface antigen is the HA glycoprotein, and we used pan-FeMV HA gene primer sets to detect additional viruses (e.g., FeMV^US2^) (Technical Appendix Figure 1, panel B). An indirect immunofluorescence assay was developed to screen serum samples for FeMV-specific antibodies. Antibodies to FeMV^US1^ were detected in fixed cells expressing FeMV H glycoprotein (positive up to 1:12,800 dilution), and antibodies to FeMV^US5^ were detected in nonpermeabilized cells (positive up to 1:6,400 dilution) (Technical Appendix Figure 2). This result confirms that H glycoprotein–specific antibodies are present at high levels concurrent with the longitudinal detection of genomic RNA. A large-scale seroprevalence and cross-neutralization study is ongoing. 

We used complete genome and H gene sequences in a comprehensive phylogenetic analysis. FeMV^US1^ is closely related to viruses from Asia, highlighting the global distribution of FeMV (Figure, panel A). Compared with the sequence for the FeMV^776U^ H gene, sequences for FeMV^US1^ and FeMV^US5^ were 98% and 81% similar, and the glycoproteins were 98% and 86% identical. The complete H gene of the most divergent US strain (FeMV^US5^) clustered phylogenetically in a basal sister relationship with all other viruses from Asia and the United States (Figure, panel B), suggesting a long evolutionary association of FeMV in feline hosts.

**Figure:**
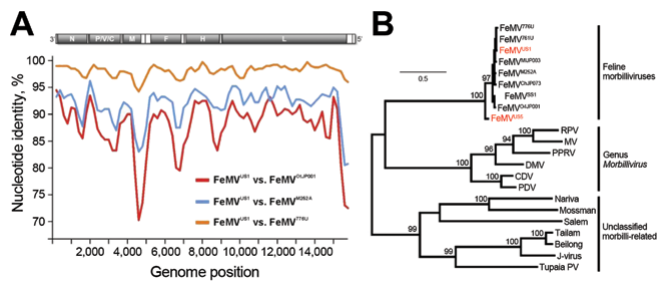
Phylogenetic analysis of feline morbillivirus (FeMV) whole genomes and hemagglutinin (H) genes collected from cats in the United States. A) Genomic sequence identity of FeMV^US1^, compared with Asian strains, performed by using SSE V1.2 software (*4*) with a sliding window of 400 and a step size of 40 nt. B) Maximum-likelihood phylogeny of the translated H gene of FeMVs, the genus *Morbillivirus*, sensu strictu, and unclassified morbilli-related viruses was determined by using MEGA5 software (*5*) and applying the Whelan-and-Goldman substitution model and a complete deletion option. Numbers at nodes indicate support of grouping from 1,000 bootstrap replicates. Scale bar indicates substitutions per site.

Ecologic surveys continue to identify novel viruses that are homologous to known paramyxoviruses in many wildlife species, including bats and rodents (*6*). Investigating closely related viruses in domestic species is warranted, given the substantial number of animals that cohabitate with humans. Switches from natural to unnatural host species can result in enhanced pathogenicity (e.g., receptor switching has caused feline panleukopenia virus to infect dogs as canine parvovirus) (*7*). Given the high degree of antigenic relatedness of morbilliviruses, understanding evolutionary origins and trajectories and conferring cross-protection through immunization are critical. Although no evidence for FeMV transmission to humans or other animals exists, the propensity for noncanonical use of signaling lymphocytic activation molecule 1F1 (CD150) should be investigated because epizootic transmission of morbilliviruses can occur (*8*).

The detection of FeMV sequences in a clinically healthy animal after 15 months is a novel and surprising observation but is consistent with the known propensity for morbilliviruses to persist in vivo (*9*). All known morbilliviruses cause acute infections, and the typical long-term clinical manifestations occur in the central nervous system, not the urinary system (*1*). These observations should prompt additional research because the prevalence of CKD in cats is high and because CKD decreases the quality of life of affected animals and is the ultimate cause of death for approximately one third of cats (*10*).

**Technical Appendix.** Methods for the molecular and serologic detection of feline morbillivirus in clinical samples.
