# Supplementary material for: Chronic Infection of Domestic Cats with Feline Morbillivirus, United States
Source: Emerg Infect Dis. 2016 Apr;22(4):760–2. doi: 10.3201/eid2204.151921 (PMC4806961; doi:10.3201/eid2204.151921)
Supplement: Supplementary file 1 — Technical Appendix. Methods for the molecular and serologic detection of feline morbillivirus in clinical samples. [file 15-1921-Techapp-s1.pdf]

Article DOI: http://dx.doi.org/10.3201/eid2204.151921

# Chronic Infection of Domestic Cats with Feline Morbillivirus, United States

## Technical Appendix

### Methods

Urine samples were collected by cystocentesis from 327 cats representing a combination of owned (pet) and unowned (stray) cats, as well as sick and healthy cats. Clinical information (age, sex, breed, body weight, disease status) was recorded for each cat as part of an ongoing large-scale clinical epidemiologic study to determine associations between feline morbillivirus (FeMV) carriage, seroprevalence, and disease status.

Two primers sets were used to screen each cDNA sample by PCR, a semi-nested primer set designed to amplify a conserved region of the L gene in respiroviruses, morbilliviruses, and henipaviruses (RMH) and either priFeMV$^{US1-L}$, priFeMV$^{US5-L}$, or priFeMV$^{panUS-L}$, which amplify a conserved region of the FeMV L gene (Technical Appendix Table 1). A third primer set, feACTβ, was used to amplify a region of feline β-actin, a ubiquitous cell maintenance gene, as a positive control (Technical Appendix Table 1). PCR was performed for each primer set using *Taq* DNA Polymerase (Thermo Fisher Scientific, Grand Island, NY, USA) according to manufacturer’s instructions. Reaction mixtures (50 μL) containing 2 μL of cDNA and 1 μL (0.2 μmol/L final concentration) of each primer, were denatured at 94°C for 2 minutes, followed by 40 cycles of 94°C for 15 seconds, 50°C for 30 seconds, and 72°C for 30 seconds, and a final extension at 72°C for 7 minutes. For the RMH primer set, 2 μL of the first PCR reaction was used as a template for a nested PCR using the same conditions as before and using 1 μL each (0.2 μmol/L final concentration) of RMH F2 and R primers. Samples (8 μL) were separated by 1% (w/v) DNA agarose gel electrophoresis to verify the presence of correct sized amplicons. Correct sized amplicons (positive samples) were either purified using QIAquick PCR purification kit (QIAGEN, Hilden, Germany) or were gel-extracted and purified using QIAquick gel extraction kit (QIAGEN); the positive samples were then sequenced (Genewiz, Inc., South Plainfield, NJ, USA) with the same primers (1.67 μmol/L final concentration) used to amplify the target region

Total RNA extractions from urine used for cDNA synthesis were performed in a clean room, using dedicated pipettes, reverse transcriptase, primers, and plasticware. PCRs were set up using different pipettes, dedicated DNA-dependent DNA polymerases and primers. Reverse-transcriptase–negative controls were always included to demonstrate that amplicons were not attributable to contamination. No tube that might contain an FeMV amplicon was ever opened in the clean room. All DNA gel electrophoresis was performed in a separate laboratory on a different floor. Even though we do not present the data in this manuscript, the sequences of all FeMV samples obtained are unique, further demonstrating specificity.

**Technical Appendix Table 1.** Sequences of FeMV-specific primers sets used to generate amplicons from cDNA after reverse transcription

| Gene | Name | Sequence (5′→3′) | Amplicon |
|---|---|---|---|
| L gene | RMH F1 | TCITTCTTTAGAACITTYGGNCAYCC | 610 bp |
| | RMH R | CTCATTTTGTAIGTCATYTTNGCRAA | |
| | RMH F2 | GCCATATTTTGTGGAATAATHATHAAYGG | 493 bp |
| | RMH R | CTCATTTTGTAIGTCATYTTNGCRAA | |
| | US1+ | TATCTGGAGAAGGGTTAACATCTG | 405 bp |
| | US1– | TCAGCTATTACTTGACAAGCCCTC | |
| | US5+ | ATCATGCATCCGCTGTAATTAG | 357 bp |
| | US5– | AGACTATATGAGAGATTGAACTC | |
| | panUS+ | ATGTTTTATGCCATTAAGTC | 460 bp |
| | panUS– | GTTGAGAATTATCTTTAGGTACAC | |
| | US1-rt+ | GCCAGAGAGAATTGAGTCTATATC | 103 bp |
| | US1-rt– | TGGYTTACCATTGAACAAGACTTTG | |
| | US1-rtPr | FAM-CAACAAAAATCGCTTGGCTAATGACCCYAA-BHQ1 | Real-time probe |
| HA gene | US1+ | AGGATTTAGTATTTAGAAGAGG | 2,077 bp |
| | US1– | GTACTTCCGGGTATAGAATATC | |
| | US5+ | GCAATACTATCCTATACACATG | 2,155 bp |
| | US5– | AGATAACTATAGGACTGTTGAG | |
| | panUS+ | GGTCAAGGAACATATAGTAG | 619 bp |
| | panUS– | GTATAATTGTAAGGTGGTATT | |

*RMH, respiroviruses, morbilliviruses, and henipaviruses.

**Technical Appendix Table 2.** Primers used for the reverse transcription and amplification of the complete genome of feline morbillivirus (FeMV) from total RNA in 11 amplicons

| Amplicon (bp) | cDNA synthesis | cDNA primer sequence (5′→3′) | Forward primer | Sequence (5′→3′) | Reverse primer | Sequence (5′→3′) | Kit* |
|---|---|---|---|---|---|---|---|
| Leader (382) | priAdaptor-dT17 | GAC TCG AGT CGA CAT CGA TTT TTT TTT TTT TTT TT | priAdaptor-dT17 | GAC TCG AGT CGA CAT CGA TTT TTT TTT TTT TTT TT | priFeMV$^{US1}$ 347– | AAT TGA CCT GGA GAT TCG AC | P |
| N (820) | priFeMV$^{US1}$ 3,541– | GAC ACC ATG AAG AAG TAG ATA C | priFeMV$^{776}$ 38+ | TGT GAC CTA TTC TAA CGA CAA G | priFeMV$^{776}$ 858– | CAA TAT CAC AAA TCA TTT CAG C | T |
| N-P (2,344) | priFeMV$^{US1}$ 754+ | CTG AGA TTG AGC AAA GGA TGG C | priFeMV$^{US1}$ 754+ | CTG AGA TTG AGC AAA GGA TGG C | priFeMV$^{US1}$ 3,098– | CTG ATG GTT GAT GTG CTT GCA TG | P |
| P-M (465) | priFeMV$^{US1}$ 3,541– | GAC ACC ATG AAG AAG TAG ATA C | priFeMV$^{776}$ 3,076+ | CAA GAA CGA AAC ATC TGC AAT C | priFeMV$^{US1}$ 3,541– | GAC ACC ATG AAG AAG TAG ATA C | T |
| M (1,516) | priFeMV$^{US1}$ 754+ | CTG AGA TTG AGC AAA GGA TGG C | priFeMV$^{US1}$ 3,293+ | CAT GCA TTA TAG GTT GTA ATT G | priFeMV$^{US1}$ 4,809– | CTG AGT TAG ACA GGC CCT AGA C | P |
| F-H (4,216) | priFeMV$^{US1}$ 4,697+ | GAT TCA TTA AAG TTA GAT TCT TG | priFeMV$^{US1}$ 4,697+ | GAT TCA TTA AAG TTA GAT TCT TG | priFeMV$^{US1}$ 8,913– | GTA CTT CCG GGT ATA GAA TAT C | P |
| L (1,553) | priFeMV$^{US1}$ 4,697+ | GAT TCA TTA AAG TTA GAT TCT TG | priFeMV$^{US1}$ 8,744+ | GCC ATT TTC AAT TTA ATA AGC TG | priFeMV$^{US1}$ 10,297– | ACT CAT TTC TGA CAG GTG AC | P |
| L (1,908) | priFeMV$^{776}$ 7,007+ | CAA AGA TTC TAG CCG GTA C | priFeMV$^{US1}$ 10,261+ | TGT TTT ATG CCA TTA AGT CTA G | priFeMV$^{776}$ 12,169– | CCC TTA GTA GTG TCA AGC ATA C | P |
| L (1,754) | priFeMV$^{776}$ 7,007+ | CAA AGA TTC TAG CCG GTA C | priFeMV$^{776}$ 12,118+ | ACT TAA TAA TCC CTA GAG CAG | priFeMV$^{US1}$ 13,872– | GGA TTT GTT CTC CTC TCA TTA TC | P |
| L (2,159) | priFeMV$^{US1}$ | GAG ATC CTT | priFeMV$^{M252A}$ | GGA TGC TTA | priFeMV$^{M252A}$ | CCA GAC AAA | Q |

| Amplicon (bp) | cDNA synthesis | cDNA primer sequence (5′→3′) | Forward primer | Sequence (5′→3′) | Reverse primer | Sequence (5′→3′) | Kit* |
|---|---|---|---|---|---|---|---|
| | 10,232+ | CTG TGG ATT TAG | 13,689+ | TTT ATC TGA TC | 16,028– | GAA AGC TAT AGG | |
| Trailer (410) | priFeMV$^{US1}$ 15,525+ | GAT AGA GTG TGA TTA TCC ATC | priFeMV$^{US1}$ 15,675+ | CTT CAA CAG TTA GTC GAG CCC G | priAdaptor-dT17 | GAC TCG AGT CGA CAT CGA TTT TTT TTT TTT TTT TT | P |

*P, Phusion High-Fidelity DNA-Dependent DNA Polymerase (New England Biolabs, Ipswich, MA, USA); T, Taq DNA-Dependent DNA Polymerase (Thermo Fisher Scientific, Grand Island, NY, USA); Q, Q5 High-Fidelity DNA-Dependent DNA Polymerase (New England Biolabs).

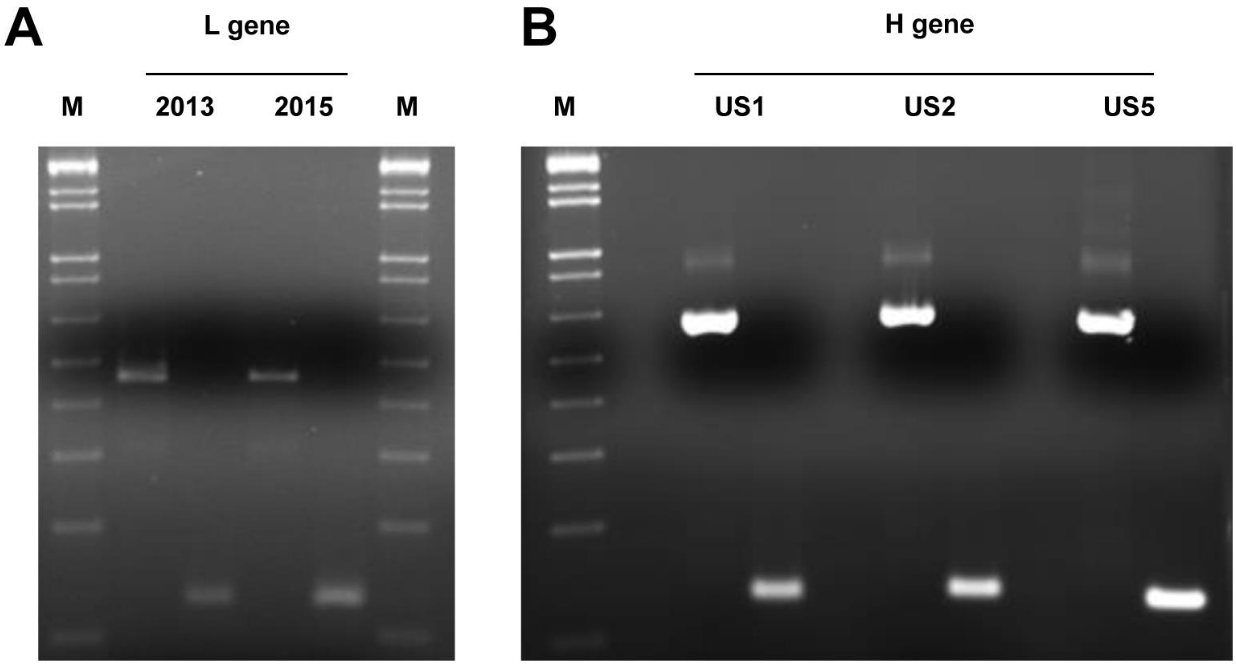


**Technical Appendix Figure 1.** Detection of feline morbillivirus (FeMV) genomic RNA in urine samples and FeMV-specific antibodies in serum from domestic cats. A) Longitudinal samples from 2013 and 2015 were collected from animal 0213, total RNA was isolated, cDNA was generated by reverse transcription, and partial L gene amplicons (460 bp) were obtained using priFeMV$^{US}$panL (+/-). B) Generation of partial HA gene amplicons (619 bp) from cDNA produced from total RNA isolated from urine samples of animals 0213 (US1), 0382 (US2), and 0122 (US5). Feline β-actin amplicons (127 bp) were generated as positive controls, and a 1 kb Plus Ladder (Thermo Fisher Scientific, Grand Island, NY, USA) was used for size markers. Reverse transcriptase (RT)–negative controls were routinely included to demonstrate specificity and ensure that amplicons were not obtained from cross-contaminating DNA (data not shown).

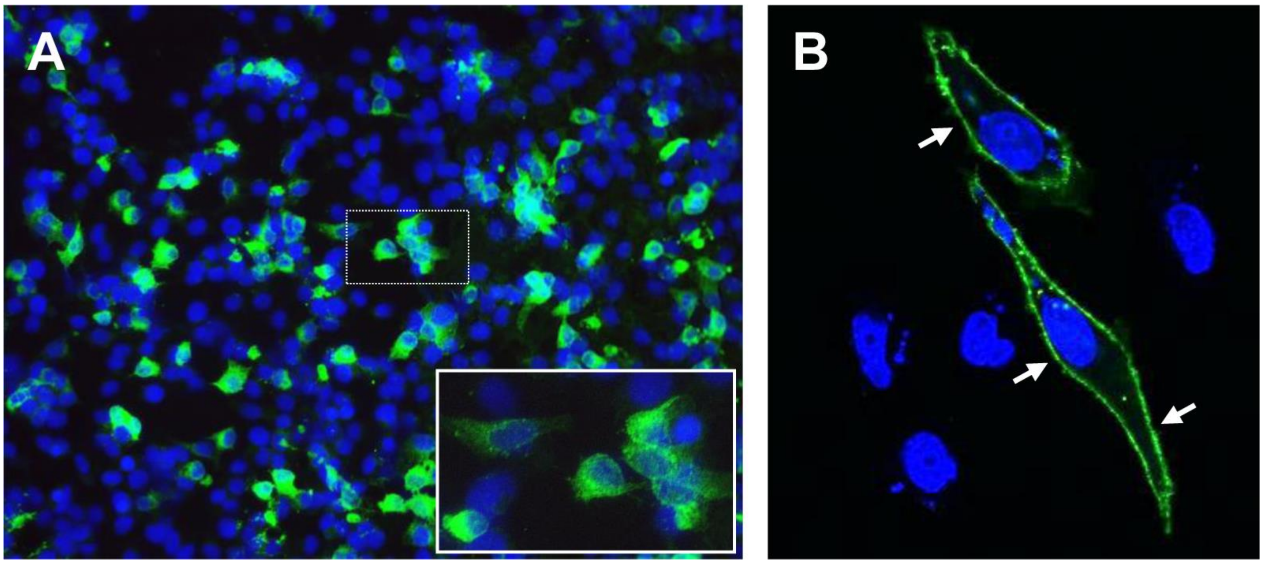


**Technical Appendix Figure 2.** Detection of feline morbillivirus (FeMV)–specific antibodies in serum from domestic cats. Vero cells were transfected with a plasmid expressing the hemagglutinin glycoprotein of $FeMV^{US1}$ (A) or $FeMV^{US5}$ (B). Cells were fixed and permeabilized (A) or nonpermeabilized (B), and indirect immunofluorescence was performed to detect the glycoproteins in polyclonal serum (1:800 dilution) from animal 0213 (A) and animal 0122 (B). Bound antibodies were detected by using Alexa Fluor 488 goat anti-cat secondary immunoglobulin G (Thermo Fisher Scientific, Grand Island, NY, USA). Analogous to the hemagglutinin glycoprotein in other morbilliviruses, the FeMV hemagglutinin glycoprotein localized to the endoplasmic reticulum and Golgi apparatus, and the protein was exclusively detected at the outer surface membrane in nonpermeabilized cells (indicated by arrows). Nuclei were counterstained with 4',6-diamidino-2-phenylindole, and fluorescence was visualized with confocal scanning laser microscopy by using a Leica SP5 Acousto-Optical Beam Splitter system (Leica Microsystems, Inc., Buffalo Grove, IL, USA).
